# tBN-CSDI: a time-varying blue noise-based diffusion model for time-series imputation

**DOI:** 10.1093/bioadv/vbaf225

**Published:** 2025-09-22

**Authors:** Graham Bishop, Tong Si, Isabelle Luebbert, Noor Al-Hammadi, Haijun Gong

**Affiliations:** Department of Mathematics and Statistics, Saint Louis University, Saint Louis, MO 63103, United States; Department of Health and Clinical Outcomes Research, Saint Louis University, Saint Louis, MO 63104, United States; Department of Mathematics and Statistics, Saint Louis University, Saint Louis, MO 63103, United States; Department of Health and Clinical Outcomes Research, Saint Louis University, Saint Louis, MO 63104, United States; Department of Mathematics and Statistics, Saint Louis University, Saint Louis, MO 63103, United States

## Abstract

**Motivation:**

Missing data imputation remains a critical challenge in high-dimensional time-series data analysis, where traditional methods often struggle to capture complex nonlinear dependencies inherent in sequential data. Diffusion-based generative models have shown state-of-the-art performance by modeling the conditional distribution of missing values given observed data. However, these models typically rely on isotropic white noise during training, which can obscure important frequency-dependent correlations that are crucial for accurate imputation.

**Results:**

To address the limitations of conventional imputation methods, we propose a novel approach called time-varying blue noise-based conditional score-based diffusion model (tBN-CSDI). By modulating the noise schedule according to the frequency characteristics of the data, tBN-CSDI improves the recovery of subtle, high-frequency temporal patterns that are often overlooked by existing techniques. Experimental results on both healthcare and single-cell RNA-seq datasets show that tBN-CSDI consistently outperforms existing imputation methods, achieving over a 30% reduction in imputation error under high data sparsity. These findings underscore tBN-CSDI’s potential as a robust and effective solution for imputing sparse and noisy time-series data. We further discuss its practical applications in improving change-point detection and gene regulatory network inference, demonstrating its broader utility in biomedical and biological research.

**Availability and implementation:**

The computer code and data for the proposed method are available on GitHub: https://github.com/gbishop345/tBN-CSDI.

## 1 Introduction

Time-series data analysis plays a crucial role in diverse fields including healthcare (Poyraz and Marttinen 2023), meteorology (Han *et al.* 2021), finance (Hsieh *et al.* 2011, Xiang *et al.* 2022), and omics research (Sha *et al.* 2024). However, missing values are a pervasive issue in real-world time-series datasets, arising from factors such as technological limitations, sensor failures, privacy constraints, and human error. In certain applications, most notably in single-cell RNA sequencing (scRNA-seq), missing rates can exceed 50% due to the intrinsic sparsity of the data and technical dropouts during the sequencing process (Xu *et al.* 2020). A time-series dataset with excessive missing values can severely compromise the reliability and accuracy of data analysis, potentially leading to incorrect forecasting of critical processes such as disease progression, patient outcomes, environmental patterns, and gene expression dynamics. Therefore, accurate imputation of missing values in high-dimensional time-series data is a critical and challenging task across many domains.

Various techniques have been developed to address missing value imputation in time-series data, and several comprehensive reviews have categorized these approaches in detail. To maintain focus and brevity, we highlight only the most widely used and state-of-the-art imputation methods that are directly relevant to and used for comparison in this work. Traditional deterministic imputation techniques, such as linear interpolation, mean or median substitution, regression-based models, ARIMA (Bartholomew 1971), and K-Nearest Neighbors (KNN) (Batista *et al.* 2002), usually generate a single fixed estimate for each missing value. Among statistical learning-based methods, multivariate imputation by chained equations (MICE) (Buuren and Groothuis-Oudshoorn 2011) is a widely adopted technique that performs iterative imputation by modeling each variable conditionally on others through alternating regression models. Matrix factorization approaches, such as SoftImpute (Hastie *et al.* 2015) and TIDER (Liu *et al.* 2023), estimate missing entries by decomposing the incomplete data matrix into low-rank components. While effective in some structured settings, these methods rely on simplified assumptions, such as linearity, low-rank structure, or local similarity, which limit their ability to model the complex nonlinear dependencies and dynamic temporal patterns frequently found in real-world time-series datasets. In contrast, recent approaches based on deep learning, attention mechanisms, and probabilistic modeling can generate multiple plausible imputations that reflect uncertainty and better capture long-range dependencies.

Recent advances in generative models, including variational autoencoders (VAEs) (Kingma 2013), generative adversarial networks (GANs) (Goodfellow *et al.* 2014), and denoising diffusion probabilistic models (DDPMs) (Ho *et al.* 2020), have significantly advanced deep learning-based imputation techniques. Unlike traditional statistical approaches, these generative models can capture complex, high-dimensional data distributions, making them particularly effective for imputing missing values in structured datasets such as time series. By conditioning on the observed values, generative models are capable of reconstructing missing entries in a statistically coherent and flexible manner. Among VAE-based methods, GP-VAE (Fortuin *et al.* 2020) learns a latent probabilistic structure from fully observed sequences and imputes missing values by sampling from the learned latent space. GAN-based frameworks have achieved considerable success in a variety of domains, including missing data imputation. By training a generator and a discriminator in an adversarial setup, the generator can learn to approximate complex data distributions and generate realistic imputations that are consistent with the observed data. For static datasets, GAN-based imputation methods such as GAIN (Yoon *et al.* 2018), and its single-cell RNA-seq extension sc-fGAIN (Si *et al.* 2023), have demonstrated strong performance by using convolutional neural networks and f-divergence objectives to generate realistic and biologically meaningful imputations. In time-series applications, recurrent neural networks (RNNs), such as Long Short-Term Memory (LSTM) and Gated Recurrent Units (GRUs), have been integrated into GAN frameworks to effectively model temporal dependencies and capture complex, high-dimensional sequential distributions, enabling more accurate imputation of missing values over time. Several GAN-based models have been developed for time-series imputation, leveraging the strengths of both adversarial training and recurrent architectures. For example, GRUI (Luo *et al.* 2018) couples modified GRUs with Wasserstein-GANs in a two-stage architecture; E2-GAN (Luo *et al.* 2019) and ImputeGAN (Qin and Wang 2023) integrate autoencoders within adversarial training pipelines; and tf-biGAIN (Liu *et al.* 2025) introduces a bidirectional framework based on f-divergence to enhance the accuracy of structured data imputation. Recently, self-attention based methods, such as SAITS (Du *et al.* 2023), GLIMA (Suo *et al.* 2020), and MBGAN (Shukla and Marlin 2021), have been introduced to effectively capture temporal dependencies for time-series imputation.

Score-based diffusion models, including DDPMs (Ho *et al.* 2020) and score matching with Langevin dynamics (Song and Ermon 2019), have emerged as another powerful class of deep generative framework. These models iteratively transform noise into data samples through a learned reverse diffusion process and have achieved state-of-the-art performance in domains such as image generation (Rombach *et al.* 2022), inpainting (Lugmayr *et al.* 2022), and structured data imputation (Tashiro *et al.* 2021, Chen *et al.* 2023). Among these, the conditional score-based diffusion model for imputation (CSDI) (Tashiro *et al.* 2021) was one of the first to adapt diffusion models specifically for time-series imputation. CSDI extends DDPMs by conditioning the diffusion process on observed data and learning to reconstruct missing values through a masked training procedure. Variants such as CSDI_T (Zheng and Charoenphakdee 2022), MissDiff (Ouyang *et al.* 2023), and DiffPuter (Zhang *et al.* 2025) have further refined this framework by incorporating tabular modeling and Expectation-Maximization inference. Despite their success, these models typically rely on isotropic Gaussian white noise, which distributes energy uniformly across frequencies and lacks temporal structure. This can suppress subtle but important high-frequency dynamics in sequential data, limiting the model’s ability to accurately reconstruct fine-grained temporal dependencies.

In this work, we propose a time-varying blue noise-based conditional score-based diffusion model (tBN-CSDI) for data imputation. Our method introduces structured blue noise into the diffusion process, enhancing the model’s ability to capture high-frequency variations while preserving temporal autocorrelations. To the best of our knowledge, this is the first diffusion-based imputation approach to systematically incorporate blue noise for reconstructing complex, temporally structured data. We evaluate our model on both healthcare and single-cell RNA sequencing (scRNA-seq) datasets and demonstrate its superior performance under high sparsity conditions.

## 2 Methods

Let $X=\{x_{1:K,1:L}\}\in R^{K\times L}$ denote a multivariate time-series dataset with *K* features and *L* time points. A binary mask matrix $M=\{m_{1:K,1:L}\}\in {\left\{0,1\right\}}^{K\times L}$ indicates whether each entry in X is observed or missing, where


$$m_{k,l}=\{\begin{array}{cc} 1, & \text{if}\;x_{k,l}\;\text{is}\;\text{observed},\\ 0, & \text{if}\;x_{k,l}\;\text{is}\;\text{missing}. \end{array}$$

Let $s=\{s_{1:L}\}\in R^{L}$ denote the time intervals between consecutive time points. Our goal is to learn a conditional distribution over the missing values $X^{\text{mis}}$ given the observed entries $X^{\text{obs}}$, along with the mask and timing information:


$$p(X^{\text{mis}}|X^{\text{obs}},M,s).$$

Most diffusion-based imputation methods rely on white Gaussian noise due to its mathematical tractability. However, white noise does not capture the temporal dependencies inherent in real-world time-series data. To address this limitation, we propose a tBN-CSDI. Our approach extends the DDPMs and builds upon the CSDI. For completeness, brief reviews of DDPM and CSDI are provided in the Appendices A and B. In the following, we detail how blue noise is integrated into the diffusion-based imputation process.

### 2.1 Blue noise generation

Blue noise is characterized by a frequency spectrum that emphasizes high-frequency components while suppressing low-frequency ones, thereby reducing long-range correlations and promoting spatial uniformity. These properties have been widely utilized in computer graphics applications (Zhang *et al.* 2020, Ahmed *et al.* 2022), including image dithering (Georgiev and Fajardo 2016) and photorealistic rendering (Wolfe *et al.* 2022). In diffusion-based models, blue noise provides a structured alternative to white Gaussian noise, which lacks temporal correlation and can obscure low-frequency trends in time-series data. By preserving underlying temporal dependencies, blue noise enables more accurate and coherent reconstruction during both the forward and reverse diffusion processes, ultimately improving imputation performance.

Generating white noise is straightforward as it involves drawing independent and identically distributed samples from a standard Gaussian distribution with zero mean and identity covariance. In contrast, generating blue noise is more complex, as it requires introducing structured correlations that suppress low-frequency components. Ulichney’s void-and-cluster algorithm (Ulichney 1999), combined with simulated annealing, is a well-known method for generating blue noise. It produces binary masks (composed of 0 s and 1 s) that exhibit blue noise characteristics and effectively suppress low-frequency components in the spatial domain. Although computationally expensive for large grids, this method yields high-quality masks with well-defined blue noise spectra.

Huang’s work (Huang *et al.* 2024) introduced a Cholesky-based method to generate blue noise with a specified covariance matrix that encodes desired frequency-domain correlations. Given a symmetric positive-definite covariance matrix Σ, Cholesky decomposition is applied to factorize it as $\Sigma =LL^{⊤}$, where L is a lower-triangular matrix. A standard multivariate Gaussian vector ε∼N(0,I) is then sampled, and the structured/correlated blue noise vector is constructed as:


$$\varepsilon_{\text{blue}}=L\varepsilon .$$

The Cholesky-based method (Huang *et al.* 2024) efficiently generates correlated noise when a predefined or learned covariance matrix is available, enabling control over spectral characteristics. In contrast, Ulichney’s void-and-cluster algorithm (Ulichney 1999) produces high-quality blue noise masks without requiring a covariance matrix, effectively suppressing low-frequency components. By integrating these two approaches, we combine computational efficiency with enhanced spectral fidelity.

Algorithm 1 outlines a hybrid blue noise generation procedure that integrates two complementary methods: Ulichney’s void-and-cluster algorithm and a Cholesky-based sampling technique. This hybrid approach combines the perceptual quality of Ulichney’s simulated annealing-based mask generation with the computational efficiency of Cholesky-based sampling. First, a set of high-quality blue noise masks is generated using Ulichney’s method. These masks are then used to estimate a covariance matrix that captures the desired spectral characteristics of blue noise. The covariance matrix is subsequently decomposed via Cholesky factorization to enable efficient sampling of new blue noise realizations that preserve the target frequency structure. In the Algorithm 1, the annealing rate r∈(0,1) controls the rate of temperature decay in the simulated annealing process; we set r=0.995 to ensure gradual cooling. The parameter *S* denotes the maximum number of sweeps (iterations) per mask, governing how long the algorithm searches for an optimal binary mask.

Algorithm 1 Blue Noise Generation via Simulated Annealing and Cholesky Decomposition

**Input:** Sequence length *L*, features *K*, number of masks *N*, annealing parameters $\left(T_{\text{init}},T_{\text{final}},r,S\right)$, ideal radial spectrum $s_{\text{ideal}}$, energy threshold ϵ

**Output:** Cholesky factor $L_{\text{blue}}$ and Blue noise $\varepsilon_{\text{blue}}$

1: Compute radial bins $r_{\text{bins}}$ for shape L×K

2: **for**  i=1 to *N* **do** 

3:   Initialize $M_{i}\in {\left\{0,1\right\}}^{L\times K}$ with 50% sparsity

4:   Set temperature $T\leftarrow T_{\text{init}}$

5:   Initialize energy E for $M_{i}$

6:   **for**  s=1 to *S* **do** 

7:     Propose a swap between one 1 and one 0 entry in $M_{i}$ to get $M_{i}^{\prime }$

8:     Compute radial spectrum $s_{i}^{\prime }$ by binning 2D Fourier magnitude using $r_{\text{bins}}$

9:     Compute energy: $E^{\prime }\leftarrow \text{MSE}(s_{i}^{\prime },s_{\text{ideal}})$

10:     $\Delta E\leftarrow E^{\prime }-E$

11:     **if**  ΔE<0  **or**  rand()<exp(−ΔE/T)  **then** 

12:       Accept: $M_{i}\leftarrow M_{i}^{\prime }$, $E\leftarrow E^{\prime }$

13:     **end if** 

14:     T←r·T

15:     **if** acceptance rate too low **then**  T←1.2·r·T

16:     **end if** 

17:     **if**  $T<T_{\text{final}}$  **or**  E<ϵ  **then** 

18:       **break** 

19:     **end if** 

20:   **end for** 

21: **end for** 

22: Stack all masks into matrix: $M\in R^{N\times (L\cdot K)}$

23: Center the masks: $M_{c}=M-\bar{M}$

24: Compute covariance: $C=\frac{1}{N-1}M_{c}^{⊤}M_{c}$

25: Compute Cholesky factor: $L_{\text{blue}}=\text{Cholesky}(C)$

26: Sample z∼N(0,I) and compute $\varepsilon_{\text{blue}}=L_{\text{blue}}z$

27: **return**  $L_{\text{blue}}$ and $\varepsilon_{\text{blue}}$

### 2.2 Time-varying blue noise-based CSDI

Using pure blue noise at every diffusion step in CSDI may overemphasize high-frequency components and neglect important low-frequency structures necessary for accurate imputation. To address this, we propose a time-varying noise scheduling strategy that interpolates between white noise and blue noise throughout the reverse diffusion process. This strategy is motivated by the reconstruction approach in Huang *et al.* (2024). In the early reverse diffusion stages, when recovering global, low-frequency patterns is critical, we use a higher proportion of Gaussian white noise to encourage modeling of the overall structure. As the process advances, we gradually increase the proportion of blue noise to enhance local detail and spatial coherence.

To balance the complementary strengths of both noise types, similar to Huang *et al.* (2024), we define a time-varying noise schedule. At each diffusion step *t*, the added noise ${\tilde{\epsilon }}_{t}$ is formulated as a linear combination of isotropic Gaussian white noise $\epsilon_{\mathrm{white}}$ and structured blue noise $\epsilon_{\mathrm{blue}}$. The resulting time-varying noise is expressed as


(1) $${\tilde{\epsilon }}_{t}=\gamma_{t}\epsilon_{\mathrm{white}}+(1-\gamma_{t})\epsilon_{\mathrm{blue}},$$

where the noise blending coefficient $\gamma_{t}\in [0,1]$ controls the trade-off between modeling global and local structure. The choice of $\gamma_{t}$ is empirical; a linear schedule was used in Huang *et al.* (2024), while Chen (2023) proposed a gamma-based schedule parameterized by three hyperparameters. Building on these approaches, our empirical results suggest that a sigmoid-based variant of the gamma schedule provides superior performance for imputation tasks by enabling a smoother and more adaptive transition between Gaussian white and blue noise. Specifically, we define the noise blending coefficient $\gamma_{t}$ as:


(2) $$\gamma_{t}=\sigma \left(\gamma_{\text{start}}+(\gamma_{\text{end}}-\gamma_{\text{start}}){\left(\frac{t}{T}\right)}^{\gamma_{\tau }}\right),$$

where *T* is the total number of diffusion steps, σ(·) denotes the sigmoid function, and the parameters $\gamma_{\text{start}}$, $\gamma_{\text{end}}$, and $\gamma_{\tau }$ control the initial value, final value, and curvature of the schedule, respectively.

Algorithm 2 and Algorithm 3 outline the detailed training and imputation procedures, respectively, for the proposed tBN-CSDI. During the training procedure illustrated in Algorithm 2, the tBN-CSDI model is trained to predict the noise added during the forward diffusion process, as defined in Equation (B.4). Unlike standard approaches that rely solely on Gaussian white noise, tBN-CSDI incorporates a blended noise signal composed of both white noise and blue noise. This blended noise is constructed using a time-dependent blending schedule, which enables the model to learn from a more structured noise spectrum across diffusion steps. The training objective is to optimize the neural network $\epsilon_{\theta }$ by minimizing the conditional diffusion loss, formulated as the mean squared error between the actual and predicted noise:


(3) $$\underset{\theta }{\min}E_{x_{0}∼q(x_{0}),\tilde{\epsilon },\;t}\left[{\left\|\tilde{\epsilon }-\epsilon_{\theta }(x_{t}^{\text{mis}},t|x_{0}^{\text{obs}})\right\|}_{2}^{2}\right].$$

Algorithm 2 Training Procedure for tBN-CSDI

**Input:** Distribution of training data $q(x_{0})$; γ parameters; number of iterations $N_{\text{iter}}$; diffusion steps *T*; precomputed Cholesky factor $L_{\text{blue}}$ in Algorithm 1

**Output:** Trained denoising model $\epsilon_{\theta }$

1: **for**  i=1 to $N_{\text{iter}}$  **do** 

2:   Sample $x_{0}∼q(x_{0})$ and t∼U({0,…,T−1})

3:   Partition $x_{0}$ into $x_{0}^{\mathrm{obs}}$ and $x_{0}^{\mathrm{mis}}$ components

4:   Sample Gaussian white noise $\epsilon_{\text{white}}∼N(0,I)$

5:   Generate blue noise: $\epsilon_{\text{blue}}=L_{\text{blue}}\epsilon_{\text{white}}$

6:   Compute blending coefficient $\gamma_{t}$ using Equation (2)

7:   Generate time-varying noise using Equation (1)

$$\tilde{\epsilon }=\gamma_{t}\cdot \epsilon_{\text{white}}+(1-\gamma_{t})\cdot \epsilon_{\text{blue}}$$

8:   Diffuse the missing component:

$$x_{t}^{\text{mis}}=\sqrt{{\bar{\alpha }}_{t}}\cdot x_{0}^{\;\text{mis}}+\sqrt{1-{\bar{\alpha }}_{t}}\cdot \tilde{\epsilon }$$

9:   Predict noise: $\hat{\epsilon }=\epsilon_{\theta }(x_{t}^{\text{mis}},t|x_{0}^{\text{obs}})$

10:   Update parameters θ by solving Equation (3).

11: **end for** 

After training the diffusion model, imputation is performed using the learned denoising network $\epsilon_{\theta }$. During the imputation process illustrated in Algorithm 3, the tBN-CSDI model also employs the time-varying blended noise $\tilde{\epsilon }$ defined in Equation (1), ensuring alignment between training and inference. This consistency improves the model’s ability to iteratively refine missing values in a coarse-to-fine manner. At each reverse diffusion step, the model updates the estimate of the missing entries using the dynamics defined in Equations (B.2) and (B.3), but with the modified noise term $\tilde{\epsilon }$, thereby preserving spectral structure throughout the imputation trajectory.

Algorithm 3 Imputation Procedure for tBN-CSDI

**Input:** A data sample $x_{0}$, trained denoising model $\epsilon_{\theta }$, diffusion steps *T*.

**Output:** Imputed missing values ${\hat{x}}_{0}^{\mathrm{mis}}$

1: Partition $x_{0}$ into $x_{0}^{\mathrm{obs}}$ and $x_{0}^{\mathrm{mis}}$

2: Initialize $x_{T}^{\mathrm{mis}}∼N(0,I)$

3: **for**  t=T−1 to 0 **do** 

4:   Sample white noise $\epsilon_{\text{white}}$ and blue noise $\epsilon_{\text{blue}}$

5:   Compute blending coefficient $\gamma_{t}$ using Equation (2)

6:   Generate time-varying noise $\tilde{\epsilon }$ using Equation (1)

7:   Compute $x_{t-1}^{\mathrm{mis}}=\frac{1}{\sqrt{\alpha_{t}}}(x_{t}^{\mathrm{mis}}-\frac{\beta_{t}}{\sqrt{1-{\bar{\alpha }}_{t}}}\epsilon_{\theta }(x_{t}^{\mathrm{mis}},t|x_{0}^{\mathrm{obs}}))$

8:   **if**  t>0  **then** 

9:     Compute $\sigma_{t}=\sqrt{\frac{1-{\bar{\alpha }}_{t-1}}{1-{\bar{\alpha }}_{t}}\cdot \beta_{t}}$

10:     Add noise: $x_{t-1}^{\mathrm{mis}}\leftarrow x_{t}^{\mathrm{mis}}+\sigma_{t}\cdot \tilde{\epsilon }$

11:   **end if** 

12: **end for** 

13: Return ${\hat{x}}_{0}^{\mathrm{mis}}=x_{0}^{\mathrm{mis}}$

**Figure 1.**
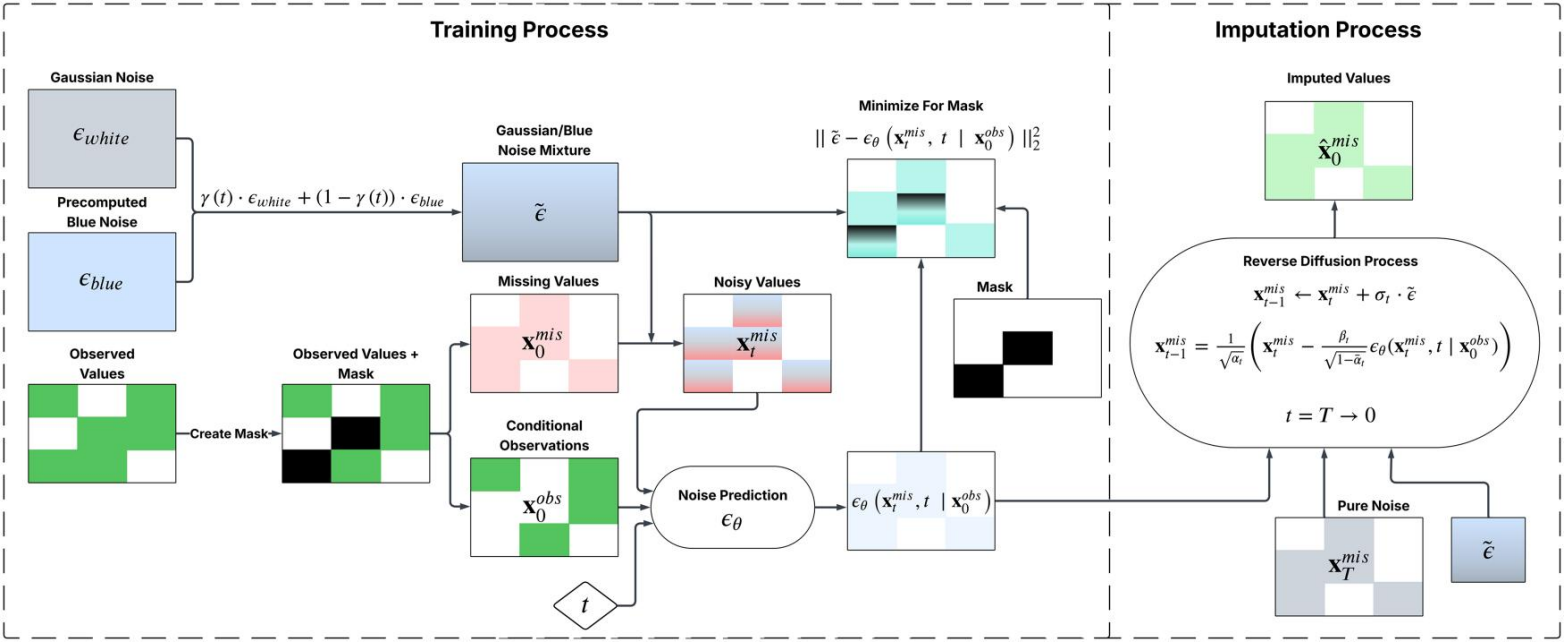
Architecture of the time-varying blue noise-based conditional score-based diffusion model (tBN-CSDI) for time-series imputation, which encompasses time-varying blue noise generation, diffusion model training, and imputation processes.

Figure 1 presents a flowchart of the tBN-CSDI framework for imputing missing values in time-series data. It includes three main components: time-varying blue noise generation, diffusion model training, and imputation, corresponding to Algorithms 1 through 3. The framework blends the Gaussian white noise and blue noise over time to form a dynamic, time-varying noise schedule. During training, this schedule emphasizes global structure recovery in early steps via white noise, and local detail refinement in later steps via blue noise. The same noise schedule is used during imputation to ensure consistency. By interpolating between these noise types, tBN-CSDI effectively combines their complementary strengths, enabling accurate and robust multivariate time-series imputation.

## 3 Results

In this section, we implement our tBN-CSDI to impute missing values in time-series data and compare its performance against several state-of-the-art imputation methods.

### 3.1 Dataset

To evaluate the general applicability and robustness of our proposed method, tBN-CSDI, for time-series imputation, we conducted experiments on three real-world datasets: (i) the PhysioNet multivariate healthcare dataset (Silva *et al.* 2012), (ii) a small single-cell RNA-seq (scRNA-seq) dataset of THP-1 human myeloid leukemia cells (Kouno *et al.* 2013), and (iii) a large scRNA-seq dataset of mouse embryonic stem cells (mESCs) (Hayashi *et al.* 2018). These datasets have been widely used as benchmarks in previous imputation and network inference studies (Luo *et al.* 2018, Yoon *et al.* 2018, Liu *et al.* 2025, Wang *et al.* 2025b).

It is important to note that time-series scRNA-seq data, such as the THP-1 dataset, differ structurally from conventional time-series datasets like PhysioNet. In traditional time-series data, repeated measurements are collected from the same subjects over time. In contrast, scRNA-seq data are cross-sectional at each time point: individual cells are lysed and sequenced, so the same cells cannot be followed longitudinally. Nevertheless, time-series scRNA-seq data can still approximate the dynamic progression of cellular states by capturing snapshots of heterogeneous cell populations over time.

### 3.2 Evaluation metrics and experimental setup

Model performance was first quantitatively evaluated using two point-wise metrics: root mean squared error (RMSE) and mean absolute error (MAE), which together provide a comprehensive assessment of imputation accuracy across different methods and datasets. Specifically, for time-course scRNA-seq data, evaluation metrics such as RMSE and MAE assess how well the model captures population-level temporal trends rather than subject-specific dynamics, given that measurements are derived from different cells at each time point. Lower RMSE and MAE values indicate better imputation performance.

We will also evaluate the probabilistic accuracy of the imputed distributions using the Continuous Ranked Probability Score (CRPS) (Matheson and Winkler 1976), which is widely used in probabilistic time-series analysis (Tashiro *et al.* 2021). CRPS measures the discrepancy between the predicted cumulative distribution function (CDF) *F* and the observed value *x*, defined as:


$$\text{CRPS}(F,x)=\int_{-\infty }^{\infty }{\left(F(z)-1\{z\geq x\}\right)}^{2}dz,$$

where 1{z≥x} is the Heaviside step function. Lower CRPS values indicate a better match between the predicted distribution and the true observation, reflecting both sharpness and calibration of the probabilistic imputation.

To evaluate robustness under varying levels of data sparsity, we introduced artificial missingness by randomly masking observed values at five different rates: 10%, 30%, 50%, 70%, and 90%. Prior to modeling, each feature (e.g., gene) was independently normalized using only the observed values to prevent information leakage. In each experiment, we compared our proposed tBN-CSDI method with several state-of-the-art imputation approaches, including vanilla CSDI, K-Nearest Neighbors (KNN), SoftImpute, and four additional methods implemented in Tashiro *et al.* (2021): V-RIN, BRITS, GLIMA, and RDIS. Due to the stochastic nature of both tBN-CSDI and vanilla CSDI, we performed five independent runs for each missingness level and reported the mean and standard deviation of the results. For deterministic baselines, results were averaged over five seeded test splits and corresponding masking patterns.

### 3.3 Imputation of PhysioNet healthcare data

We evaluated our model on the PhysioNet Challenge dataset (Silva *et al.* 2012), a widely used benchmark for multivariate time-series imputation. This dataset contains clinical records from ∼4000 ICU patients, each covering the first 48 h post-admission and including physiological variables such as age, weight, heart rate, glucose levels, and other vital signs, sampled irregularly over time. For our experiments, we selected 35 variables with consistent availability across patients. The dataset is highly sparse, with roughly 80% missing values, making it particularly challenging for imputation methods. To ensure robust and unbiased evaluation, we partitioned the data into training (70%), validation (10%), and test (20%) sets under a fivefold cross-validation scheme. Performance was assessed using ground-truth masks, and each experiment was repeated five times to compute the mean absolute error (MAE) and root mean squared error (RMSE), along with their standard errors.

Table 1 reports the mean MAE and RMSE for various imputation methods under different missing rates, introduced by randomly masking observed values. Our results show that the proposed tBN-CSDI consistently outperforms vanilla CSDI as well as widely used deterministic methods, KNN and SoftImpute, across all levels of missingness. Furthermore, since prior work by Tashiro *et al.* (2021) demonstrated that vanilla CSDI outperforms several established methods, including V-RIN, BRITS, GLIMA, and RDIS, under various missing rates (10%, 50%, and 90%), it follows that our tBN-CSDI also surpasses all these baselines. Table 1 also reports the runtime of tBN-CSDI and vanilla CSDI methods. Our results indicate that the imputation time of both methods remains relatively stable regardless of the missing rate, suggesting that sparsity has minimal impact on computational cost. Note that MAE, RMSE, and CRPS were calculated concurrently, so their runtime measurements are identical.

**Table 1.** Comparison of MAE, RMSE, and runtime (seconds) using different imputation methods and missing rates on the PhysioNet dataset.^a^

Metric	Missing rate	tBN-CSDI	CSDI	KNN	SoftImpute	Time (tBN)	Time (CSDI)
	0.1	0.185 (0.003)	0.217 (0.002)	0.375 (0.002)	0.404 (0.007)	2154.0 (5.37)	2066.0 (7.28)
	0.3	0.216 (0.001)	0.257 (0.002)	0.447 (0.003)	0.480 (0.004)	2152.4 (8.09)	2066.9 (7.42)
MAE	0.5	0.252 (0.004)	0.305 (0.002)	0.495 (0.002)	0.566 (0.003)	2152.1 (10.55)	2068.9 (8.38)
	0.7	0.299 (0.004)	0.370 (0.002)	0.570 (0.002)	0.651 (0.004)	2151.5 (9.62)	2064.7 (8.81)
	0.9	0.391 (0.008)	0.485 (0.007)	0.697 (0.003)	0.702 (0.003)	2146.3 (6.32)	2069.8 (7.01)
	0.1	0.459 (0.060)	0.502 (0.025)	0.593 (0.007)	0.602 (0.015)	2154.0 (5.37)	2066.0 (7.28)
	0.3	0.462 (0.015)	0.640 (0.013)	0.714 (0.009)	0.710 (0.008)	2152.4 (8.09)	2066.9 (7.42)
RMSE	0.5	0.606 (0.033)	0.679 (0.005)	0.779 (0.004)	0.809 (0.007)	2152.1 (10.55)	2068.9 (8.38)
	0.7	0.601 (0.028)	0.730 (0.005)	0.855 (0.007)	0.896 (0.008)	2151.5 (9.62)	2064.7 (8.81)
	0.9	0.693 (0.024)	0.837 (0.008)	0.950 (0.006)	0.947 (0.007)	2146.3 (6.32)	2069.8 (7.01)

a Each experiment was repeated five times, with the mean and standard error (in parentheses) reported.

To further compare with the vanilla CSDI, we also computed CRPS, where lower CRPS values indicate better performance. Prior work by Tashiro et al. demonstrated that vanilla CSDI achieves the lowest CRPS among several baseline methods (V-RIN, BRITS, GLIMA, and RDIS). As shown in Table 2, our results indicate that tBN-CSDI consistently outperforms the vanilla CSDI across all missing rates, suggesting that tBN-CSDI would also surpass the other baseline methods not recomputed in this study.

**Table 2.** Comparison of CRPS using tBN-CSDI and CSDI on PhysioNet data.^a^

Missing rate	tBN-CSDI	CSDI
0.1	0.201 (0.003)	0.237 (0.002)
0.3	0.234 (0.001)	0.281 (0.002)
0.5	0.273 (0.004)	0.328 (0.012)
0.7	0.324 (0.004)	0.404 (0.002)
0.9	0.423 (0.010)	0.527 (0.007)

a Each experiment was repeated five times, with the mean and standard error (in parentheses) reported.

### 3.4 Imputation of small-scale THP-1 scRNA-seq data

Our second experiment used a small-scale single-cell RNA sequencing (scRNA-seq) dataset profiling 120 distinct monocytic THP-1 human myeloid leukemia cells at each of eight time points (0, 1, 6, 12, 24, 48, 72, and 96 h) following stimulation with 12-myristate 13-acetate (PMA), totaling 960 cells (Kouno *et al.* 2013). The THP-1 dataset captures temporal gene expression profiles for 45 genes associated with cellular differentiation. Each cell represents a sample with gene expression measured at a specific time point. As is typical in scRNA-seq experiments, the cells measured at each time point are different, providing cross-sectional snapshots of the underlying dynamic process. Following a setup similar to the PhysioNet experiment, we partitioned the data into training (60%), validation (20%), and test (20%) sets using fixed random seeds. Gene expression values were independently normalized using non-missing training data. To simulate different sparsity levels, we randomly masked observed values at varying missing rates. Imputation performance was evaluated using MAE, RMSE, and CRPS, averaged over five repeated runs per setting with standard errors reported.

Table 3 summarizes the mean MAE, RMSE, and runtime of various imputation methods on the THP-1 scRNA-seq dataset across different missing rates. Our tBN-CSDI method consistently achieves the lowest MAE across all levels of missingness, significantly outperforming both the vanilla CSDI and deterministic baselines. In particular, tBN-CSDI reduces the error by approximately 35%–40% compared to vanilla CSDI. Compared to KNN and SoftImpute, the performance gap is even more pronounced: the MAE of tBN-CSDI is about one-third of KNN and SoftImpute in some settings. Similarly, the RMSE results further validate the superior performance of our proposed tBN-CSDI method in imputing scRNA-seq data. Across all missing rates, tBN-CSDI consistently achieves the lowest RMSE, outperforming vanilla CSDI, KNN, and SoftImpute by substantial margins. At the highest missing rate of 90%, tBN-CSDI attains an RMSE of 0.587, compared to 0.962 for vanilla CSDI and over 1.0 for the other baselines. Notably, tBN-CSDI maintains stable performance across all missing rates, while the baseline methods deteriorate as sparsity increases. This highlights tBN-CSDI’s robustness and reliability under extreme data sparsity. Runtime analysis shows that tBN-CSDI is comparable to vanilla CSDI and remains largely unaffected by the degree of missingness, consistent with the observations from the PhysioNet experiments.

**Table 3.** Comparison of MAE, RMSE, and runtime (seconds) using different imputation methods and missing rates on the THP-1 scRNA-seq data.^a^

Metric	Missing rate	tBN-CSDI	CSDI	KNN	SoftImpute	Time (tBN)	Time (CSDI)
	0.1	0.403 (0.005)	0.634 (0.010)	0.867 (0.019)	0.769 (0.016)	122.6 (4.97)	124.0 (1.53)
	0.3	0.391 (0.002)	0.642 (0.008)	0.876 (0.013)	0.775 (0.012)	123.2 (7.34)	122.9 (1.69)
MAE	0.5	0.390 (0.005)	0.641 (0.007)	0.916 (0.006)	0.784 (0.008)	119.2 (0.89)	125.3 (2.91)
	0.7	0.397 (0.007)	0.650 (0.003)	0.989 (0.009)	0.793 (0.006)	118.8 (0.75)	124.6 (2.13)
	0.9	0.412 (0.003)	0.672 (0.004)	0.858 (0.004)	0.792 (0.002)	119.8 (0.63)	124.1 (0.98)
	0.1	0.578 (0.009)	0.918 (0.010)	1.110 (0.029)	0.995 (0.025)	122.6 (4.97)	124.0 (1.53)
	0.3	0.564 (0.008)	0.932 (0.005)	1.138 (0.018)	1.020 (0.015)	123.2 (7.34)	122.9 (1.69)
RMSE	0.5	0.558 (0.009)	0.931 (0.008)	1.197 (0.012)	1.028 (0.012)	119.2 (0.89	125.3 (2.91)
	0.7	0.563 (0.009)	0.931 (0.003)	1.303 (0.018)	1.038 (0.009)	118.8 (0.75)	124.6 (2.13)
	0.9	0.587 (0.006)	0.962 (0.003)	1.143 (0.012)	1.040 (0.005)	119.8 (0.63)	124.1 (0.98)

a Each experiment was repeated five times, with the mean and standard error (in parentheses) reported.

### 3.5 Imputation of large-scale mESC scRNA-seq data

Our third experiment was conducted on a large-scale scRNA-seq dataset profiling gene expression dynamics in mouse embryonic stem cells (mESCs) undergoing differentiation into primitive endoderm cells (Hayashi *et al.* 2018). The dataset contains expression measurements for thousands of genes across 421 single cells, sampled at five distinct time points (0, 12, 24, 48, and 72 h). Due to the memory constraints of existing imputation methods when applied to high-dimensional data, we restricted our analysis to a representative subset of 100 randomly selected genes. The original dataset contains a substantial proportion of zero values, which we treated as missing, yielding a large and sparse matrix that provides a challenging benchmark for evaluating scalability and robustness. This setting allows us to rigorously assess the ability of our proposed tBN-CSDI model to impute missing values in multivariate time-series gene expression data. The experimental setup and procedures were kept identical to those used in the THP-1 scRNA-seq analysis to ensure comparability.

Table 4 presents the MAE, RMSE, and runtime results for imputation performance on the large-scale mESC scRNA-seq dataset. Consistent with previous findings from the THP-1 dataset, our proposed tBN-CSDI method consistently achieves the lowest errors across all conditions. Specifically, the MAE for tBN-CSDI remains stably around 0.45 across different missingness levels, while the RMSE is consistently near 0.59. In contrast, other methods such as CSDI, KNN, and SoftImpute report significantly higher MAE values between 0.7 and 0.9, and RMSE values often exceeding 0.95. These results demonstrate the robustness and scalability of tBN-CSDI in handling high-dimensional, sparse single-cell data. Even under extreme sparsity (up to 90%), tBN-CSDI maintains strong and consistent performance, demonstrating its effectiveness and generalization capability across a wide range of data sparsity scenarios. Runtime analysis further shows that both tBN-CSDI and vanilla CSDI are insensitive to the degree of missingness, consistent with the earlier observations.

**Table 4.** Comparison of MAE, RMSE, and runtime (seconds) using different imputation methods and missing rates on mESC scRNA-seq data.^a^

Metric	Missing rate	tBN-CSDI	CSDI	KNN	SoftImpute	Time (tBN)	Time (CSDI)
	0.1	0.456 (0.005)	0.727 (0.005)	0.907 (0.020)	0.787 (0.021)	113.0 (1.59)	107.9 (0.46)
	0.3	0.448 (0.005)	0.730 (0.006)	0.954 (0.019)	0.785 (0.010)	113.9 (0.42)	110.0 (0.44)
MAE	0.5	0.451 (0.001)	0.725 (0.002)	0.964 (0.015)	0.787 (0.015)	116.2 (2.56)	110.3 (0.94)
	0.7	0.449 (0.005)	0.727 (0.003)	0.941 (0.018)	0.785 (0.011)	116.7 (5.35)	110.5 (1.76)
	0.9	0.452 (0.008)	0.732 (0.001)	0.818 (0.015)	0.783 (0.011)	114.2 (1.11)	111.7 (1.39)
	0.1	0.590 (0.006)	0.950 (0.005)	1.156 (0.030)	0.974 (0.038)	113.0 (1.59)	107.9 (0.46)
	0.3	0.587 (0.007)	0.966 (0.008)	1.228 (0.022)	0.993 (0.016)	113.9 (0.42)	110.0 (0.44)
RMSE	0.5	0.592 (0.004)	0.961 (0.002)	1.248 (0.015)	0.993 (0.022)	116.2 (2.56)	110.3 (0.94)
	0.7	0.590 (0.008)	0.970 (0.003)	1.221 (0.026)	0.992 (0.013)	116.7 (5.35)	110.5 (1.76)
	0.9	0.594 (0.013)	0.976 (0.004)	1.052 (0.020)	0.995 (0.015)	114.2 (1.11)	111.7 (1.39)

a Each experiment was repeated five times, with the mean and standard error (in parentheses) reported.

To further assess the probabilistic imputation performance, we also computed the CRPS values for both the small-scale THP-1 dataset and the larger, high-dimensional mESC dataset. As shown in Table 5, tBN-CSDI achieves CRPS values of ∼0.40 on THP-1 and 0.44 on mESC, significantly lower than the corresponding values of around 0.60 and 0.70 obtained by the vanilla CSDI model across all missing rates. These consistently lower CRPS values demonstrate that tBN-CSDI not only improves pointwise accuracy (as shown in prior MAE and RMSE evaluations) but also enhances the quality of its probabilistic predictions. Lower CRPS indicates that our model provides more reliable uncertainty estimates, which is critical for downstream biological analyses where decision-making may depend on the confidence of imputations. Moreover, the consistent improvements across both datasets highlight the robustness and scalability of tBN-CSDI in handling diverse single-cell RNA-seq scenarios, from small datasets to large-scale, high-dimensional time-course experiments, and underscore its potential as a reliable and effective imputation method for single-cell transcriptomic data.

**Table 5.** Comparison of CRPS using tBN-CSDI and CSDI on THP-1 and mESC scRNA-seq data.^a^

	THP-1		mESC	
Missing rate	tBN-CSDI	CSDI	tBN-CSDI	CSDI
0.1	0.408 (0.002)	0.623 (0.006)	**0.443 (0.005)**	0.695 (0.006)
0.3	0.394 (0.002)	0.618 (0.006)	**0.438 (0.002)**	0.698 (0.004)
0.5	0.393 (0.003)	0.620 (0.005)	**0.444 (0.001)**	0.703 (0.002)
0.7	0.397 (0.004)	0.627 (0.003)	**0.445 (0.002)**	0.708 (0.003)
0.9	0.401 (0.002)	0.643 (0.002)	**0.446 (0.003)**	0.710 (0.001)

a Each experiment was repeated five times, with the mean and standard error (in parentheses) reported.

## 4 Discussion

In this work, we propose tBN-CSDI, a novel conditional score-based diffusion model that incorporates time-varying blue noise for time-series imputation. To the best of our knowledge, this is the first imputation framework to systematically integrate structured, frequency-aware noise into the diffusion process. By replacing conventional isotropic Gaussian white noise with dynamically evolving blue noise, tBN-CSDI effectively captures frequency-dependent temporal patterns. This approach addresses key limitations of existing diffusion models and provides a robust and flexible solution for imputing sparse and noisy time-series data. Time-varying blue noise is generated by integrating Ulichney’s void-and-cluster algorithm, based on simulated annealing, with a Cholesky decomposition-based sampling technique, enabling the creation of structured noise that evolves smoothly over time. By interpolating between white noise and blue noise over time, tBN-CSDI captures both global and local structures, effectively modeling frequency-dependent correlations and fine-grained temporal dynamics in time-series data. This approach preserves the probabilistic integrity of the diffusion framework and leads to improved generalization across varying sparsity levels. Extensive experiments on benchmark healthcare and single-cell RNA sequencing datasets show that tBN-CSDI consistently outperforms vanilla CSDI and other state-of-the-art imputation methods. Our model achieves over a 30% reduction in imputation error, delivering significantly lower RMSE, MAE, and CRPS values, particularly under high data sparsity. These results demonstrate the effectiveness of incorporating frequency-sensitive noise structures into diffusion-based learning and suggest promising directions for spectral-aware modeling in time-series imputation, particularly for the sparse and noisy single-cell data.

Importantly, tBN-CSDI is highly flexible and can be readily extended to handle not only time-series data but also static and conditionally structured datasets beyond temporal domains. Building on its effectiveness in robust time-series imputation, a promising direction for future work is to integrate tBN-CSDI with change-point and anomaly detection algorithms (Si *et al.* 2024, Wang *et al.* 2025a) to improve the identification of dynamic shifts and irregularities in temporal patterns. Accurate imputation of missing values in sparse and noisy time-series data using tBN-CSDI can significantly improve the sensitivity and precision of change-point and anomaly detection, enabling a more reliable identification of key temporal events, such as stages of disease progression or critical changes in cellular states. Additionally, another promising direction for future research is the integration of tBN-CSDI with network inference algorithms (Wang *et al.* 2025b) to reconstruct gene regulatory networks from time-series scRNA-seq data following imputation. Because missing values and dropout effects can significantly distort gene–gene relationships, using tBN-CSDI as a preprocessing step can enhance data quality and lead to more accurate, biologically meaningful network reconstructions. Together, these directions will extend the utility of tBN-CSDI beyond imputation, establishing it as a core component in an integrated framework for comprehensive temporal and regulatory analysis.

## Data Availability

The computer code and data are available on GitHub: https://github.com/gbishop345/tBN-CSDI

## Appendix

### A Denoising diffusion probabilistic models

In this appendix, we briefly review denoising diffusion probabilistic models (DDPMs) (Ho *et al.* 2020). Given data sampled from an unknown distribution $q(x_{0})$, DDPMs aim to learn a model distribution $p_{\theta }(x_{0})$ that approximates $q(x_{0})$. This is achieved through a two-stage generative process: a forward diffusion process, which gradually adds noise to the data, and a reverse denoising process, which learns to recover the original data distribution by progressively removing the noise.

Let $x_{t}$ for t=1,…,T denote a sequence of latent variables in the same sample space X as the observed variable $x_{0}$. In the forward process, Gaussian noise is incrementally added to $x_{0}$ via a Markov chain, gradually transforming it into a nearly isotropic Gaussian-like variable $x_{T}$ over *T* time steps. The joint distribution of this forward process is defined as:

(A.1)
$$\begin{array}{cc} & q(x_{1:T}|x_{0}):=\prod_{t=1}^{T}q(x_{t}|x_{t-1}),\\ \text{where}, & \;q(x_{t}|x_{t-1}):=N\left(x_{t};\sqrt{1-\beta_{t}}x_{t-1},\beta_{t}I\right), \end{array}$$

and $\beta_{t}$ denotes the noise variance at time step *t*.

A closed-form expression for the distribution of $x_{t}$ conditioned directly on initial state $x_{0}$ has been derived in Ho *et al.* (2020), allowing sampling at any time step without iterating through all intermediate steps, which is given by:

(A.2)
$$q(x_{t}|x_{0})=N(x_{t};\sqrt{{\bar{\alpha }}_{t}}x_{0},(1-{\bar{\alpha }}_{t})I),$$

where ${\bar{\alpha }}_{t}=\prod_{s=1}^{t}\alpha_{s}$, and $\alpha_{s}=1-\beta_{s}$.

The reverse denoising process in DDPMs reconstructs the original data $x_{0}$ by iteratively denoising latent variables $x_{t}$, starting from Gaussian white noise $x_{T}∼N(0,I)$. This process is modeled as a Markov chain with the joint distribution:

(A.3)
$$p_{\theta }(x_{0:T}):=p(x_{T})\prod_{t=1}^{T}p_{\theta }(x_{t-1}|x_{t}).$$

When the diffusion rate $\beta_{t}$ is sufficiently small, the reverse conditional distribution $p_{\theta }(x_{t-1}|x_{t})$ can be modeled by a parameterized Gaussian distribution using a reparameterization trick implemented by a neural network (Ho *et al.* 2020):

(A.4)
$$p_{\theta }(x_{t-1}|x_{t}):=N\left(x_{t-1};\mu_{\theta }(x_{t},t),\Sigma (x_{t},t)\right),$$

where the mean parameter is defined as:

(A.5)
$$\mu_{\theta }(x_{t},t)=\frac{1}{\sqrt{\alpha_{t}}}\left(x_{t}-\frac{\beta_{t}}{\sqrt{1-{\bar{\alpha }}_{t}}}\epsilon_{\theta }(x_{t},t)\right).$$

In addition, $x_{t}$ is generated from the forward process conditioned on $x_{0}$, that is,

(A.6)
$$x_{t}=\sqrt{{\bar{\alpha }}_{t}}x_{0}+\sqrt{\left(1-{\bar{\alpha }}_{t}\right)}\epsilon ,$$

where ϵ∼N(0,I), and $\epsilon_{\theta }(x_{t},t)$ denotes the neural network’s prediction of the noise added at timestep *t*. A fixed variance schedule, as proposed by Ho *et al.* (2020), is used for $\Sigma (x_{t},t)$, and is defined as:

(A.7)
$$\Sigma (x_{t},t)=\Sigma_{t}=\frac{(1-{\bar{\alpha }}_{t-1})\beta_{t}}{1-{\bar{\alpha }}_{t}}I.$$

Previous studies (Ho *et al.* 2020) have demonstrated that training the reverse process in DDPMs involves minimizing the evidence lower bound (ELBO), which can be simplified to a denoising score matching objective. Specifically, the training reduces to minimizing the prediction error between the true Gaussian noise and the noise predicted by a neural network:

(A.8)
$$\underset{\theta }{\min}E_{x_{0}∼q(x_{0}),\epsilon ∼N(0,I),t}\left[\left|\left|\epsilon -\epsilon_{\theta }(x_{t},t\right)\right|{|}_{2}^{2}\right].$$

Once trained, the diffusion model can generate realistic samples by sampling from the learned reverse process, starting from $x_{T}∼N(0,I)$, ultimately producing $x_{0}∼p_{\theta }(x_{0})$ that closely approximates the true data distribution $q(x_{0})$.

### B White noise-based diffusion model for imputation

In this appendix, we briefly review the CSDI (Tashiro *et al.* 2021), a DDPM-based framework designed for multivariate time-series imputation.

CSDI conditions the reverse diffusion process on observed data to model the conditional distribution of missing values given the observed entries. Given an input sample $x_{0}$ with missing values, the observed and missing parts are denoted by $x_{0}^{\text{obs}}$ and $x_{0}^{\text{mis}}$, respectively. The objective of CSDI is to approximate the true conditional distribution $q(x_{0}^{\text{mis}}|x_{0}^{\text{obs}})$ with a learnable parameterized model distribution $p_{\theta }(x_{0}^{\text{mis}}|x_{0}^{\text{obs}})$, where θ denotes the parameters of the neural network in the reverse diffusion process.

The forward diffusion process in CSDI follows the same formulation as in standard DDPMs: it is unconditional, and Gaussian noise is added to the complete input sample, including both observed and masked (missing) components, following a predefined noise schedule described in Equations (A.1) and (A.2). However, the reverse diffusion process is modified to be conditional on the observed values. Specifically, Equations (A.3) and (A.4) are re-formulated as follows:

(B.1)
$$p_{\theta }(x_{0:T}^{\text{mis}}|x_{0}^{\text{obs}}):=p(x_{T}^{\text{mis}})\prod_{t=1}^{T}p_{\theta }(x_{t-1}^{\text{mis}}|x_{t}^{\text{mis}},x_{0}^{\text{obs}}),$$

(B.2)
$$p_{\theta }(x_{t-1}^{\text{mis}}|x_{t}^{\text{mis}},x_{0}^{\text{obs}}):=N(x_{t-1}^{\text{mis}};\mu_{\theta }^{\prime },\Sigma_{t}),$$

where $x_{T}^{\mathrm{mis}}∼N(0,I)$, the mean $\mu_{\theta }^{\prime }$ is parameterized using a conditional denoising function $\epsilon_{\theta }$ as follows:

(B.3)
$$\begin{array}{cc} \mu_{\theta }^{\prime }=\mu_{\theta }(x_{t}^{\mathrm{mis}},t|x_{0}^{\mathrm{obs}}) & \\ =\frac{1}{\sqrt{\alpha_{t}}}\left(x_{t}^{\mathrm{mis}}-\frac{\beta_{t}}{\sqrt{1-{\bar{\alpha }}_{t}}}\epsilon_{\theta }(x_{t}^{\mathrm{mis}},t|x_{0}^{\mathrm{obs}})\right), & \end{array}$$

and the variance $\Sigma_{t}=\sigma_{t}^{2}$ remains fixed, as defined in Equation (A.7).

The training objective of CSDI is to optimize the neural network $\epsilon_{\theta }$ by minimizing the conditional diffusion loss for the imputation task, re-defined as:

(B.4)
$$\underset{\theta }{\min}E_{x_{0}∼q(x_{0}),\epsilon ∼N(0,I),t}\left[||\epsilon -\epsilon_{\theta }(x_{t}^{\text{mis}},t|x_{0}^{\text{obs}})|{|}_{2}^{2}\right].$$

Tashiro et al.’s work (Tashiro *et al.* 2021) employed a self-supervised learning approach to train the model. During training, the observed data are randomly partitioned into conditional observations $x^{\text{obs}}$ and pseudo-missing values $x^{\text{mis}}$, where the latter are masked and treated as missing to enable the model to learn to impute missing entries from available context. Once trained, imputation is performed by initializing with white Gaussian noise and iteratively applying the conditional reverse diffusion process to generate plausible time-series samples that are consistent with the observed values. Throughout the denoising process, the observed entries are explicitly preserved, ensuring that the imputed values are accurately guided by the available observations.
